# Long-term trends in rainfall and temperature using high-resolution climate datasets in East Africa

**DOI:** 10.1038/s41598-019-47933-8

**Published:** 2019-08-06

**Authors:** Solomon H. Gebrechorkos, Stephan Hülsmann, Christian Bernhofer

**Affiliations:** 1 United Nations University Institute for Integrated Management of Material Fluxes and of Resources (UNU-FLORES), 01067 Dresden, Germany; 2 Faculty of Environmental Sciences, Institute of Hydrology and Meteorology, Technische Universität Dresden, 01062 Dresden, Germany

**Keywords:** Climate-change mitigation, Statistics

## Abstract

Detecting changes in climate is a prerequisite for a better understanding of the climate and developing adaptation and mitigation measures at a regional and local scale. In this study long-term trends in rainfall and maximum and minimum temperature (T-max and T-min) were analysed on seasonal and annual time scales for East Africa. High resolution gridded rainfall (1981–2016) and temperature (1979–2010) data from international databases like the Climate Hazards Group are used. Long-term seasonal trend analysis shows a non-significant (except for small areas), decreasing (increasing) trend in rainfall in eastern (western) parts of Ethiopia and Kenya and a decreasing trend in large parts of Tanzania during the long rainy season. On the other hand, a non-significant increasing trend in large parts of the region is observed during the short rain season. With regard to annual trends, results largely confirm seasonal analyses: only a few significant trends in rainfall, but significant increasing trends in T-max (up to 1.9 °C) and T-min (up to 1.2 °C) for virtually the whole region. Our results demonstrate the need and added value of analysing climate trends based on data with high spatial resolution allowing sustainable adaptation measures at local scales.

## Introduction

Global climate has changed in recent decades and exposes a significant impact on the environment and on social and economic well-being^1,2^. The increase in air temperature and variability in precipitation are already evident in different parts of the world and their impacts on the environment (e.g., ecosystem and biodiversity) and human system are becoming evident^3^. In particular, the impact of climate variability and change on the agriculture sector is significant^4^, threatening food security and livelihoods particularly in developing countries^3^. Africa is one of the most vulnerable continents to climate change and variability due to its low adaptive capacity^5^ and a change in climate variables may lead to significant reduction in agricultural production^6^. In Africa, particularly East Africa, more than 80% of the population depends on agriculture and the income from this sector contributes about 40% to the regional GDP^7^.

The climate, particularly rainfall, in East Africa is known for its inter-annual variability, which has contributed to the devastating droughts and floods^5,8^. Several studies highlighted that the variability in rainfall in this region is linked to large-scale climate variability, including the ElNĩno Southern Oscillation (ENSO), Indian Ocean Dipole (IOD)^9,10^, and movement of the inter-tropical convergence zone (ITCZ)^11,12^. ENSO has shown multiple effects in precipitation; warming of the ocean temperature leads to an increase in rainfall and change in direction of the ITCZ^13^. IOD, on the other hand, represents the sea surface temperature variability in the tropical Indian Ocean and this change significantly affects the climate of East Africa, Indonesia, India, and some parts of Australia and Asia^14^. In general, variability in rainfall in East Africa, particularly the inter-annual variability, is modulated by large scale climate forcings and changes in sea surface temperature, which affects the rainfall amount (e.g., decrease during the long-rain season; March-May) by changing wind patterns and moisture fluxes^15^.

While a number of studies (listed above) focused on the drivers of climate variability in East Africa, the number of studies exploring long-term trends in climate variables from regional to local scale are limited^16–19^ and mostly confined to single watersheds or basins based on few station data or data with a coarser spatial resolution such as output from global and regional climate models^20,21^. Notably, studies in remote and data sparse areas, where most of the agricultural activities are happening, are limited. A study^22^ concluded that regions with poor climate information are highly vulnerable to climate change and variability, which holds for East Africa. Identifying areas with a changing in climate (e.g., rainfall and temperature) requires spatial information with higher resolution, which could help better management of the impacts. However, one of the main limitations to perform such a comprehensive study in this region is the availability of a sufficiently long-term and spatially representative climate data from the field-based meteorological stations. Even if data from few stations is available, it may not be representative for the region.

In order to address the data issue in East Africa, we have in an earlier study identified preferential data products (rainfall and maximum and minimum temperature) with high spatial and temporal resolution and accuracy (see section datasets)^23^. We evaluated multiple climate data products (satellite-based rainfall estimates, observational reanalysis hybrid, and regional climate models) using observed data from 332 stations provided by the National Meteorological Agency of Ethiopia and the global summary of the day, for Kenya and Tanzania, available at the National Climate Data Centre (NCDC, https://www7.ncdc.noaa.gov/).

Here, using the results of that study, we assess long-term trends in rainfall and maximum and minimum temperature (T-max and T-min) in East Africa, particularly Ethiopia, Kenya, and Tanzania. Such a comprehensive study covering a large part of East Africa, using climate data with high spatial resolution and on longer periods is not available as of to date. In general, the results of this study will help understand the current climate at local and regional scale and develop sustainable adaptation and mitigation measures.

## Results

### Climatology

**Figure 1:**
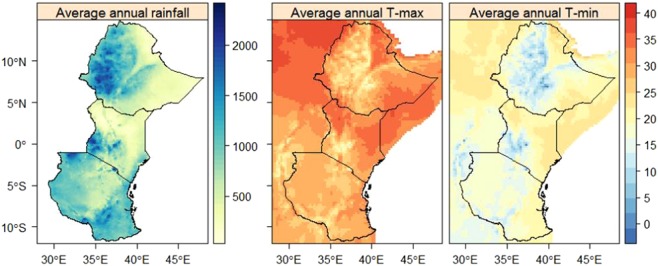
Long-term average annual rainfall (1981–2016, mm) and T-max and T-min (1979–2010, °C) of East Africa retrieved from CHIRPS and OR, respectively.

The observed long-term country-area average annual rainfall, based on the Climate Hazard Group Infrared Precipitation with Stations version 2 (CHIRPS), during 1981–2016 is higher in Tanzania (971.7 mm) than in Ethiopia (801.6 mm) and Kenya (601.1 mm) (Fig. 1). The long-term average shows a maximum rainfall record (up to 2000 mm) in the western part of Ethiopia and Kenya and south-eastern parts of Tanzania. On the other hand, lower rainfall (<500 mm) is recorded in the eastern part of Ethiopia and northern and eastern parts of Kenya. The region with lower rainfall record showed higher T-max (up to 35 °C) and T-min (up to 25 °C) records, particularly in the eastern part of the region, during 1979–2010 (based on Observational-Reanalysis Hybrid; OR). Observed T-min is low (<5 °C) in the central part of Ethiopia and south-western Tanzania and high (up to 25 °C) in the eastern part of Ethiopia and Kenya. In general, the eastern part of the region showed lower rainfall and higher temperature (T-max and T-min) records during 1981–2016 and 1979–2010, respectively.

### Seasonal trends of rainfall, T-max, and T-min

**Figure 2:**
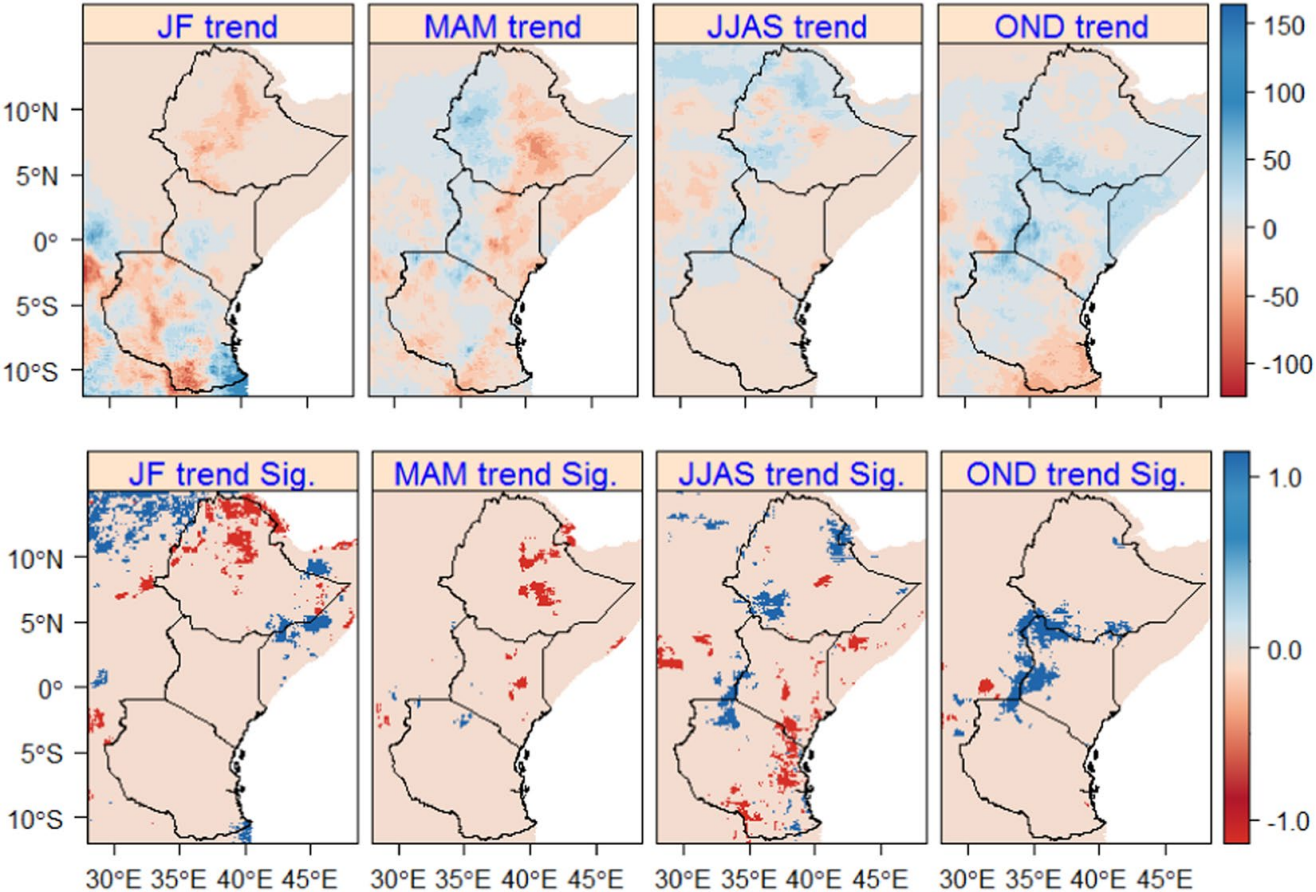
Seasonal rainfall trends (upper map) based on CHIRPS in East Africa for the period of 1981–2016. MAM and OND are the long and short rain season, respectively. Sig. (bottom map) displays the significance of the trends (P < 0.05) and 1 (blue) and -1 (red) show statistically significant increasing and decreasing trends, respectively. Statistically not significant changes are displayed in sandy-brown.

The long-term trend analysis in seasonal rainfall showed a non-significant change in large parts of the region (Fig. 2). In the northern (JF, dry season) and central-eastern (MAM, long rain season) parts of Ethiopia, a significant decreasing trend (up to −100 mm) in seasonal rainfall is observed. Additionally, in the central parts of Kenya (around Kora National reserve and Marsabit) a significant decreasing trend (up to −50 mm) is observed during MAM. During JJAS (Boreal summer season), a significant increasing trend (up to +60 mm) is observed around north-eastern and south-western (around Jimma) parts of Ethiopia and in small areas of western Kenya (around Kisumu and Bungoma) and North-western Tanzania (around Mwanza). Moreover, a significant increasing trend (up to +50 mm) during the short rain season (OND) is observed in the western parts of Kenya and southern parts of Ethiopia. In general, in the eastern parts of Ethiopia and Kenya and large parts of Tanzania a non-significant decreasing trend in rainfall is observed during MAM. On the contrary, in the western parts of Ethiopia and Kenya a non-significant increasing trend is observed during MAM. Similarly, a non-significant increasing trend is observed in large parts of the region in during OND.

**Figure 3:**
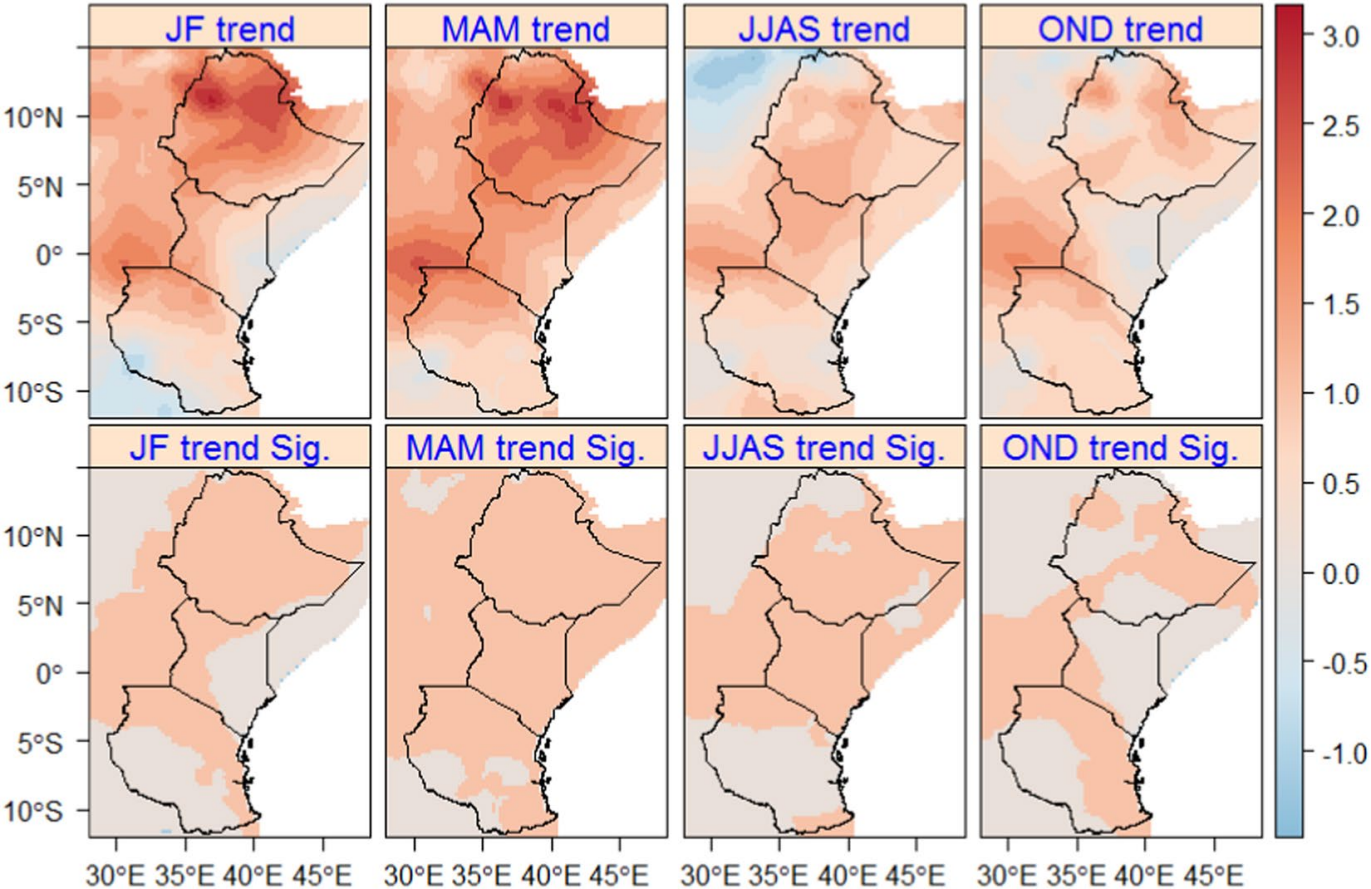
Seasonal T-max trends (upper map) based on OR in East Africa for the period of 1979–2012. Sig. (bottom map) displays the significance of the trends (P < 0.05) and 1 (coral) and -1 (light-blue) show statistically significant increasing and decreasing trends, respectively. Statistically not significant changes are displayed in gainsboro.

Unlike to the trend in seasonal rainfall which showed a significant change only in small pockets of the region, a significant increasing trend in seasonal T-max (up to +3 °C) is observed in large parts of the region (Fig. 3), particularly high during JF and MAM. During JF and OND, only in few areas of the region such as in eastern parts of Kenya and southern and south-western parts of Tanzania, a non-significant increasing trend in T-max is observed during 1979–2010. Similarly, a non-significant increasing trend in T-max is observed during MAM in large parts of Tanzania and northern parts of Ethiopia.

**Figure 4:**
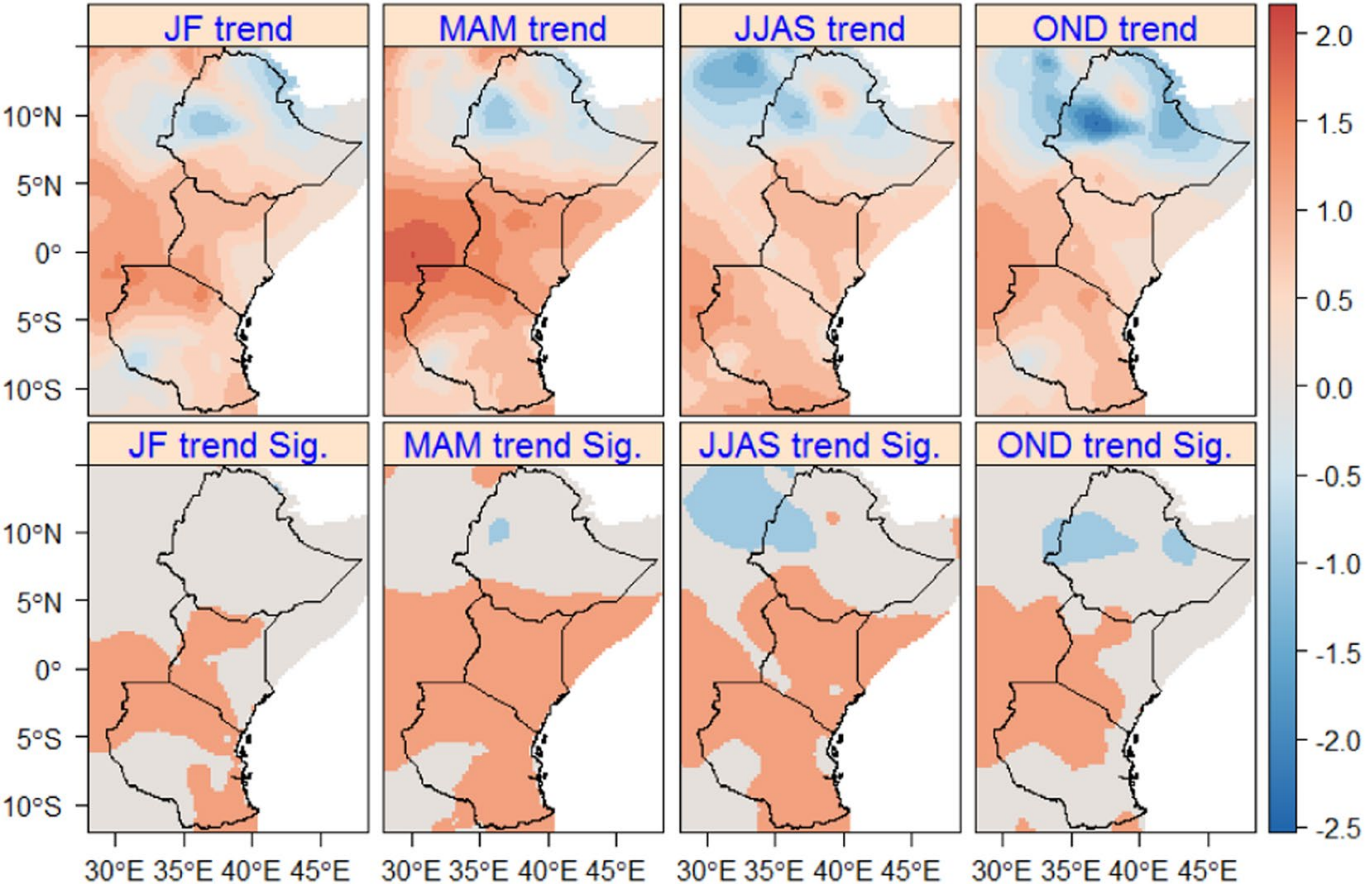
Seasonal T-min trends (upper map) based on OR in East Africa for the period of 1979–2010. Sig. (bottom map) displays the significance of the trends (P < 0.05) and 1 (coral) and -1 (light-blue) show statistically significant increasing and decreasing trends, respectively. Statistically not significant changes are displayed in gainsboro.

Similar to the change in T-max, a significant increasing trend in T-min is observed in this region, particularly in Kenya and Tanzania (Fig. 4). In the southern part of Ethiopia, the observed increasing trend (up to +1.2 °C) in T-min during MAM and JJAS is significant, which was not the case for the observed increasing trend (up to +1 °C) during JF and OND. Unlike other parts of the region, a significant decreasing trend (up to −2.5 °C) is found in the western (MAM) and eastern (JJAS and OND) parts of Ethiopia. During MAM and JJAS, T-min showed a significant increasing trend (up to +2 °C) in Kenya and Tanzania. Moreover, a significant increasing trend is observed in the north-western and northern parts of Kenya and Tanzania during JF and OND.

### Annual trends of rainfall, T-max, and T-min

**Figure 5:**
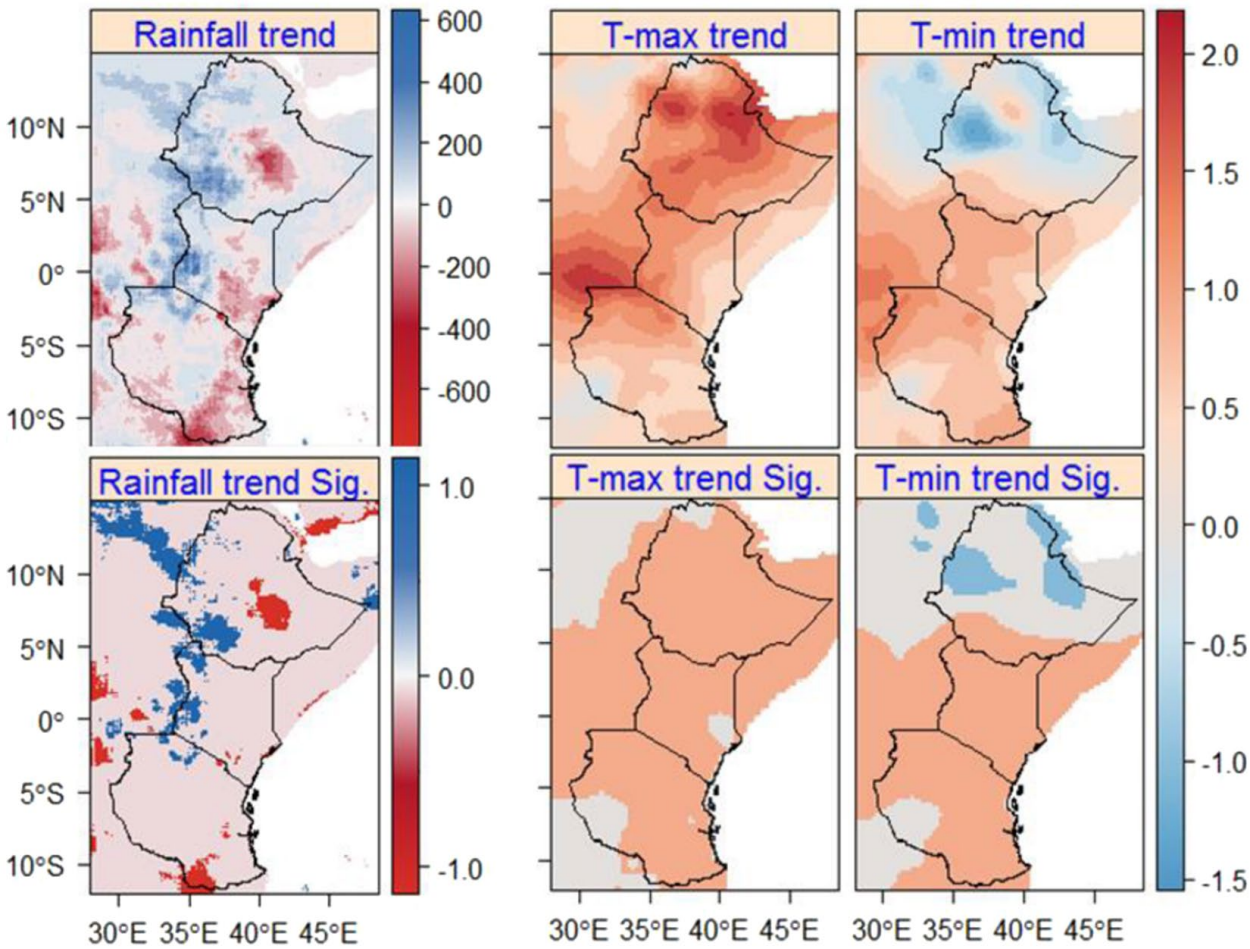
Annual rainfall (based on CHIRPS) and T-max and T-min (based on OR) trends in East Africa during the periods of 1981–2016 and 1979–2010, respectively. Sig. (bottom map) displays the significance of the trends (P < 0.05) and 1 (blue for rainfall and coral for T-max and T-min), -1 (red for rainfall and light-blue for T-max and T-min) show significant increasing and decreasing trends, respectively. Statistically not significant changes (0) are given by light-pink and gainsboro for rainfall and T-max and T-min, respectively.

With regard to annual values, results largely confirm seasonal analyses: only a few statistically significant trends in rainfall, but significant increasing trends in T-max for virtually the whole region (Fig. 5). The maximum change in T-max is found in the eastern parts of Ethiopia (up to 3 °C). Compared to Ethiopia and Kenya, the change in T-max is lower in Tanzania. Trends for T-min were regionally more diverse, significantly increasing trend (up to +1.2 °C) in Southern Ethiopia and large parts of Kenya and Tanzania and decreasing trend (up to −1.5 °C) in limited areas in the eastern and western parts of Ethiopia. In the western parts of Ethiopia (the main source of Nile Basin) and Kenya and some parts of northern Tanzania (around Musoma and Serengeti National Park) annual rainfall showed a significant increasing trend up to 600 mm. On the contrary, annual rainfall showed a significant decreasing trend in central-eastern Ethiopia (around Arsi-Bale and Harerge) and South-eastern Tanzania (around Songea). In general, the observed increasing and decreasing trends in annual rainfall in large parts of the region is not significant.

## Discussion

Based on quality controlled and best-performing high-resolution climate data products, our analysis demonstrates strong changes in rainfall, mostly non-significant, (1981–2016) and T-max and T-min (1979–2010) in the main parts of East Africa, comprising Ethiopia, Kenya, and Tanzania. While some of the general results, particularly with respect to regional averages based on GCMs and RCMs, had similarly been found in recent studies (see below), our comprehensive analysis using high-resolution climate data allows focusing on local and regional changes in much more detail than previously provided. In addition, the analysis provides a clear visualisation of the trend in rainfall, T-max, and T-min that allows identifying hotspot areas for adaptation and managing the impacts in sectors such as agriculture and water resources.

In this region, the long-term trend analysis shows a declining trend during the long rain (MAM) in the eastern parts of Ethiopia and Kenya and large parts of Tanzania. The result of the study is consistent with previous studies based on a coarse resolution of rainfall data^20,21,24^, which showed a decline in MAM rainfall in East Africa. However, using high-resolution climate data we found an increasing trend in the western parts of Ethiopia and Kenya. In addition, unlike previous studies, we found a significant decreasing trend in rainfall during MAM in the central-eastern parts of Ethiopia and Kenya. In addition, in large parts of the region an increasing trend in rainfall during the short rainy season (OND) is observed. In line with the observed trends, projections show an increase in precipitation in the western parts of Ethiopia and decrease in Kenya and Tanzania during MAM and an increase in large parts of the region during OND^25^. On a regional scale, the increase in OND rainfall is in line with the most current study^21^ based on a coarse resolution of rainfall datasets, which showed an increasing trend in rainfall during OND. However, in some areas such as the south-eastern part of Tanzania and northern parts of Ethiopia we found a non-significant decreasing trend in rainfall during OND.

During JJAS, which was less studied in this region, studies^26,27^ showed a decline in rainfall in East Africa. Our results demonstrate that this is only true for large parts of Kenya and Tanzania. However, unlike to a study based on a coarse resolution of rainfall data (ERA-Interim reanalysis data)^28^, we found, in some areas, a significant increasing trend in Ethiopia and some parts of western Kenya and north-western Tanzania. On annual time scale, previous studies based on a coarse resolution rainfall^17,29^, concluded that the change in rainfall in East Africa is not significant. However, this is only true when taking a regional average or using a coarse resolution of data, but our analysis based on high-resolution rainfall data revealed significant changes (up to ±600 mm during the period of 1981–2016) in a specific area of the region. It is noteworthy that increases were found in the comparatively wet areas, whereas the decrease was found in a comparatively dry area in Ethiopia, but a comparatively wet area in Tanzania. It was concluded that observed trends in this region largely depends on the type of dataset used^30–32^, which supports the application of a rainfall data with high spatial resolution (e.g., to detect changes at local scale) and accuracy.

The observed changes in seasonal rainfall, particularly during MAM and OND, have shown a strong impact on sectors such as agriculture, water resource, and energy. For instance, according to the report^33^, the failure in MAM rainfall in 2011 caused a significant impact on the region’s economy and more than 13 million people were victims of food insecurity. On the other hand, excessive rains in 2015 in large parts of East Africa affected about 410,000 people and 271 were killed^34^.

Regarding the temperature, the long-term trend analysis also shows a significant increasing trend in T-max (up to 1.9 °C) in Ethiopia, Kenya, and Tanzania and a significant increasing trend in T-min (up to 1.3 °C) in Kenya and Tanzania. In line with the findings of this study, other studies^5,35,36^, covering different parts of the region, showed similar increasing trends in temperature but differ in magnitude depending on the type of data. In addition to the mean change in T-max and T-min, a significant increasing trend in temperature extremes (e.g., maximum and minimum of maximum and minimum temperature) was found in East Africa^37^. In general, in most parts of Africa the mean temperature was found to have increased by more than 0.5 °C during the last 50 to 100 years^5,38–41^, while globally it increased by 0.72 °C^2^ and it is projected to increase throughout the 21 century^25^. This increase in T-max and T-min leads to increased water losses by evapotranspiration and more severe droughts might occur given the decrease in rainfall in large parts of the region. Overall, using this information, the region requires a particular attention in developing sustainable adaptation measures to minimize the impacts such as on agriculture, e.g., by the construction of water harvesting dams. Further, the fine-scaled analysis provided here may support policy-makers in selecting priority areas for implementation of adaptation measures.

## Materials and Methods

### Study area

**Figure 6:**
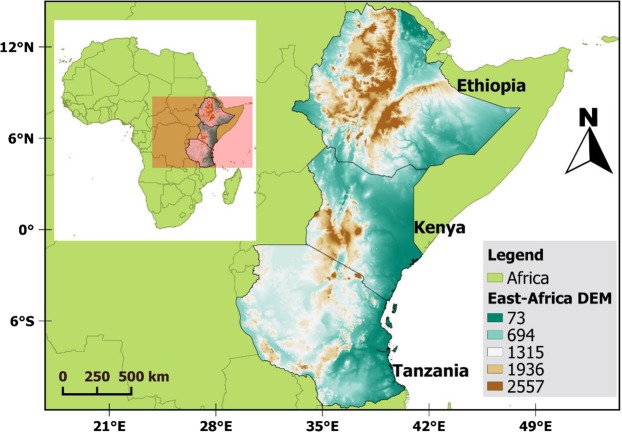
Location and elevation (DEM) map of East Africa (Ethiopia, Kenya, and Tanzania). The map is developed in Qgis 3.6^63^.

The study covers regions of East Africa, Greater Horn of Africa (GHA), particularly Ethiopia, Kenya, and Tanzania (Fig. 6). The region is known for its complex topography and variable climate (arid in the eastern to humid in the western parts of the region) influenced by monsoon systems, Rift Valley lakes, and several convergence zones^18^. Northern and southern parts of East Africa receive its higher rainfall during June-August and December-February seasons, respectively.

Areas close to the equator have a long and a short rainy season with higher rainfall records around March-May (MAM) and October-December (OND), respectively. In addition, June-September (JJAS) is typically the major rainy season in the highland parts of Ethiopia and January and February (JF) is the driest seasons with lower rainfall and higher temperature records^17^. The western part of Ethiopia is the major source of the Blue Nile River Basin and higher rainfall is observed in this area compared to other parts of the region (see Fig. 1). Higher temperature, exceeding 30 °C, is recorded in the northern and eastern parts of Ethiopia, western Kenya, and Tanzania. On the other hand, lower temperature records are observed in the highlands of Ethiopia, Kenya, and Tanzania (see Fig. 1).

### Datasets

For this study, we used gridded satellite- and reanalysis-based datasets with higher spatial and temporal resolutions following the comprehensive evaluation of multiple climate data sources for East Africa^23^. During the last decades, a number of climate data products have been developed based on climate models, remote sensing and reanalysis and their agreement with ground observations varies from product to product. According to^30,31^, application of different climate data products for trend analysis leads to different conclusions and data should be checked for its quality and accuracy (compared to station data) before application in climate studies. Therefore, in our previous study^23^, we evaluated climate data products with high spatial and temporal resolution and available for longer periods allowing for climate studies (>30 years). In order to identify the most accurate products, we used multiple approaches (e.g., station to pixel) and methods such as statistical (e.g., correlation coefficient, root mean square error, and percentage of bias), graphical (e.g., Taylor diagram), and other characteristics (e.g., wet and dry days, daily and annual totals). Finally, by comparing the products with station data on daily to monthly time scales over 21 regions of East Africa, the Climate Hazard Group Infrared Precipitation with Stations version 2 (CHIRPS) and Observational-Reanalysis Hybrid (OR) were identified for rainfall and T-max and T-min, respectively. CHIRPS is a satellite-based rainfall estimate based on daily, 5-day, and monthly time scale infrared cold cloud duration (CCD) estimates and station data. CHIRPS is a semi-global (ranging from 50 S to 50 N and all longitudes) gridded rainfall data source with a spatial resolution of 0.05° (~5 km) and it is available from 1981-present^42^. This dataset is mainly designed for monitoring of droughts and other global environmental changes in data scarce regions such as East Africa. In addition, CHIRPS allows for monitoring of climate extremes, trend analysis, and hydrological projections in a complex topography such as the Ethiopian highlands^42^. Due to its high quality and high spatial and temporal resolution and extensive quality control implemented, CHIRPS is widely used for assessing long-term variability and trends in climate^27,38,43–46^.

OR is another a widely used global^47^ and regional (Sub-Saharan Africa)^48^ dataset with high spatial and temporal resolution. OR provides multiple climate datasets (e.g., rainfall, T-max and T-min, radiation, and wind speed) that can be used as input for land surface and terrestrial models and for analysis of climate variability and change and climate extremes in data-sparse regions such as Africa^47,48^. This dataset is developed by downscaling the NCEP–NCAR (National Centers for Environmental Prediction-National Center for Atmospheric Research) reanalysis data^49^ into a spatial resolution of up to 0.1°. The hybrid data combines the Global Precipitation Climatology Project^50^ and Tropical Rainfall Measuring Mission Multisatellite Precipitation Analysis^51^ and temperature dataset from the Climate Research Unit^52^. This data is corrected for temporal inhomogeneity and biases and random errors are removed through the application of quality controlled ground station data^48^. Depending on the spatial resolution and regional coverage, OR is available at multiple time scales (1948–2012) from the Terrestrial Hydrology Research Group, University of Princeton (http://hydrology.princeton.edu/data.php). Due to its high resolution and spatial coverage, OR is widely used in hydro-climatic studies^53–55^. In this study, therefore, CHIRPS (1981–2016) for rainfall and OR (1979–2010) for T-max and T-min are used.

### Methods

The Climate Data Operator (CDO) version 1.6.4^56^ and the CMSAF (The Satellite Application Facility on Climate Monitoring) package^57^ of the open R software^58^ are used to format, rearrange, and clip datasets to a required format for the trend analysis. Both packages are further used to merge daily datasets and create grid cell based daily time series, convert units, and aggregate daily to monthly time series. In addition, the NetCDF Operator (NCO: http://nco.sourceforge.net/) is used to remove and modify dimensions and variables of the NetCDF data that interferes with packages of the R software such as CMSAF.

The monthly total rainfall and average monthly T-max and T-min created by CDO and CMSAF are divided to January-February (JF), March-May (MAM), June-September (JJAS), and October-December (OND) for seasonal trend analysis. In addition to seasonal trend analysis, the monthly data is used for annual trend analysis. To identify trends in seasonal and annual time series a linear model is fitted to each grid cell using the CMSAF package in R. Linear model (Eq. 1) and the non-parametric Mann-Kendall test (MK) are widely used to detect long-term seasonal and annual trends in multiple climate datasets^59,60^.

1 $$y=a+\beta .t$$

The slope of the regression line (β) determines the change in *y* over the change in time (t). The change is assumed to be statistically significant at a probability level of 5%. This process enables to compute the significance of a trend for each grid cell, which eases the local and large-scale analysis of climate trends. In addition, for regionally averaged time series, the significance of the trend is computed using the non-parametric Mann-Kendall test (MK)^61^. The MK test determines the presence of monotonic upward or downward tendency of data in a given time. The magnitude of change in a time series is computed by using the Sen’s slope estimator (β). The Trend^62^ package of the R software is used to compute the MK test and slope in the time series.

## Data Availability

In this study, freely available daily rainfall and temperature datasets are used following our previous study^23^. The rainfall data used in this study is the Climate Hazards Group InfraRed Precipitation with Station (CHIRPS), which is freely available at the International Research Institute (IRI) data library (https://iridl.ldeo.columbia.edu/SOURCES/.UCSB/.CHIRPS/; IRI/LDE; access date 23/November/2016). For maximum and minimum temperature, the Observational-Reanalysis Hybrid (OR) is used. OR is freely available at the Terrestrial Hydrology Research Group, Princeton University (http://hydrology.princeton.edu/data.php; access data 12/May/2016).
